# Actually Getting Some Satisfaction on the Job: Need–Supply Fit of Fundamental Motives at Work

**DOI:** 10.3389/fpsyg.2020.01740

**Published:** 2020-08-12

**Authors:** Jan Dörendahl, Christoph Niepel, Samuel Greiff

**Affiliations:** Cognitive Science & Assessment, University of Luxembourg, Esch-sur-Alzette, Luxembourg

**Keywords:** fundamental motives, need–supply fit, job satisfaction, response surface analysis, career development

## Abstract

The fit between employees’ needs and the opportunity to satisfy them in the workplace is an important predictor of job satisfaction. To make full use of this concept in career development, a fine-grained assessment of needs is necessary to allow for a straightforward interpretation. Fundamental motives provide a theoretically meaningful, self-contained framework of 16 fine-grained explicit motives, including, for instance, Social Acceptance, Curiosity, and Autonomy. Based on a series of response surface analyses in a German online sample of 723 working people, we examined the impact on job satisfaction of three different combinations of fundamental motives and their supply: exact congruence, an excess in the supply, and a shortage in the supply. For an excess in the supply, the results suggest that job satisfaction was highest for Social Acceptance, Status, Autonomy, Sex, and Retention. For a congruence of high motive levels and high supply levels, the levels of job satisfaction were highest for Curiosity, Idealism, and Social Participation. Concerning a shortage in the supply, low levels of job satisfaction were observed for Social Acceptance, Status, Sex, Retention, Curiosity, and Idealism. The results can be useful in coaching and career developments to provide information about potential sources of low job satisfaction and provide guidance to clients on how to enhance their job satisfaction.

## Introduction

The fit between individuals’ needs, desires, and preferences on the one side and the extent to which the workplace offers opportunities to satisfy these needs on the other is one of the most important predictors of job satisfaction (Kristof-Brown et al., 2005). Previous studies have demonstrated that the congruence between personal needs and the extent to which a workplace can supply what employees need positively predicts job satisfaction (Kristof-Brown et al., 2005; Krumm et al., 2013a). Higher job satisfaction, in turn, has been found to be correlated with better job performance (Judge et al., 2001), stronger identification with the organization (de Moura et al., 2009) less turnover intention (Van Dick et al., 2004; de Moura et al., 2009) and more organizational citizenship behavior (Organ and Ryan, 1995). In sum, if a job supplies what employees need, job satisfaction increases, yielding several desirable outcomes for employers and employees.

Thus, it has already been demonstrated that a fit between need and supply contributes to job satisfaction. The current study extends existing knowledge by integrating the two approaches of the *16 fundamental motives* (Havercamp, 1998; Reiss, 2004, 2008) and *response surface analysis* (Edwards and Parry, 1993). First, we examined need–supply fit on a fine-grained level as postulated by the 16 fundamental motives (Havercamp, 1998; Reiss, 2004, 2008) which represent what people consciously and ultimately strive for, as opposed to motives that are pursued for instrumental purposes. The 16 fundamental motives constitute a theoretically meaningful, self-contained framework of explicit motives, including, for instance, Social Acceptance, Curiosity, and Autonomy (see Table 1; Havercamp, 1998; Reiss, 2004, 2008; Dörendahl et al., submitted^1^). The 16 fundamental motives thus encompass what people are concerned with in their everyday lives. Nevertheless, other approaches to fundamental motives also exist, such as the framework by Kenrick et al. (2010) of six motives with a focus on evolutionary fitness. Although there is some overlap between these two frameworks (i.e., social participation/affiliation, safety/self-protection, and status motives), we elected to work with the 16 fundamental motives framework because it provides an extensive number of fine-grained motives and is frequently used by practitioners, for example, in coaching, work settings, and various other life domains (Reyss and Birkhahn, 2009).

**TABLE 1:** Descriptions of the Fundamental Motives assessed by the 16mrs.

Motive	Description
Social acceptance	Need to be accepted by other people
Status	Need to gain and maintain reputation and acquire a prominent position in society
Autonomy	Need for independence from other people
Sex	Need for a fulfilling sex life and erotic experiences
Retention	Need to build up and maintain stocks
Dominance	Need to influence people as well as processes
Family	Need to provide solicitude for one’s family. The motive mainly refers to one’s family of origin but might also include one’s partner or offspring
Physical Exercise	Need for physical activity and exercise
Food Enjoyment	Need to have pleasurable experiences involving food. This motive goes beyond the bodily need of eating
Curiosity	Need to expand one’s knowledge, gain new insights, and engage in intellectual challenges
Safety	Need for a peaceful and secure life
Idealism	Need to support disadvantaged people and improve society
Social Participation	Need for companionship
Structure	Need to organize and structure one’s environment in a simple and unambiguous manner
Morality	Need to comply with social norms that apply to society
Revenge	Need to retaliate wrongs or insults from others

*Adapted from Dörendahl et al., submitted^1^*

Previous research has supported the validity of the 16 fundamental motives. For instance, correlational analyses involving different personality frameworks have suggested that the fundamental motives reflect personality characteristics that differ from the Big Five (McCrae and Costa, 1997) and capture motivational aspects beyond the long-known and extensively investigated motives of Power, Achievement, Affiliation, Intimacy, and Fear (Heckhausen and Heckhausen, 2010; Dörendahl et al., submitted^1^). Further investigations have suggested that the fundamental motives validly predict self-reported behavior (e.g., frequency of varsity sports; Reiss et al., 2001; see also Dörendahl et al., submitted^1^) the three components of love (Sternberg, 1998; Engel et al., 2002) college students’ romantic attraction to peers with disabilities (Man et al., 2006) and school achievement (Froiland et al., 2015). Second, to exploit this extensive framework in an optimal way, we employed response surface analysis (Edwards and Parry, 1993), which allowed us to examine in detail how combinations of and discrepancies between need – as measured by the 16 fundamental motives – and supply are related to job satisfaction (Shanock et al., 2010). In summary, the results of our study provide important implications for coaching and career development processes because they uncover a potential source of low job satisfaction.

### Theoretical Background

Explicit motives include people’s self-concepts about their goals, values, personality attributes, and affective preferences (Schönbrodt and Gerstenberg, 2012). As opposed to implicit motives, explicit motives can be verbalized and can therefore be assessed with questionnaires (Brandstätter et al., 2013). Although this seems convenient at first glance, the question of which motives should actually be assessed inevitably arises, given that the number of explicit motives is basically endless. Even though on a conceptually broad level, scholars have agreed that Achievement, Power, and Affiliation are the so-called big three of motivation, no consensus has been reached about a more extensive and fine-grained framework of motives (Heckhausen and Heckhausen, 2010). To address the need for a theoretically meaningful and self-contained list of motives, Reiss and Havercamp (1996, pp. 622–625) defined four rules for identifying the motives that represent what people ultimately strive for in life, as opposed to motives that are pursued for instrumental purposes. First, the motives should be ends rather than means. That is, the motives need to be pursued for no other reason than the satisfaction of the motive itself. Second, the importance of the motive should predict the frequency and intensity of behavior targeting the satisfaction of the motive. That is, for a motive that is more important to a person, the person must show more frequent and more intense attempts to satisfy the motive in comparison with individuals for whom the motive holds only a little importance. Third and strongly connected to the previous assumption, fundamental motives reflect interindividual differences. Thus, people may differ with respect to the meaning that each of the motives holds for them and subsequently differ in the frequency and intensity of behavior that is aimed at satisfying the very same motive. Fourth and finally, fundamental motives should account for a significant amount of everyday behavior (Reiss, 1999). To this end, the motives need to be on a certain level of abstraction. If a motive is too specific, it accounts for relatively little behavior, and consequently, a large number of motives would need to be postulated to achieve a comprehensive description of human motivation (Dörendahl et al., submitted^1^).

On the basis of these four rules, Reiss and Havercamp (1996) derived a preliminary list of 25 potential fundamental motives. Using four exploratory factor analyses and one confirmatory factor analysis, they reduced the initial list to 16 fundamental motives (Havercamp, 1998; Reiss and Havercamp, 1998) that made up the final list of fundamental motives: Social Acceptance, Status, Autonomy, Sex, Retention, Dominance, Family, Physical Exercise, Food Enjoyment, Curiosity, Safety, Idealism, Social Participation, Structure, Morality, and Revenge. See Table 1 for their respective construct definitions.

Like explicit motives in general, the fundamental motives are activated by cues in the environment (Brandstätter et al., 2013). Therefore, the outcomes of any motives do not exclusively depend on the strength of the motive itself, but rather on the fit between what people desire and what the environment offers them. Consequently, for a comprehensive understanding of human motivation, researchers need to consider not only the motives themselves as personal characteristics but also the appropriate features of the environment. With respect to the workplace, as one of the major domains in the lives of people with full-time or part-time employment, the congruence of employee personality and job characteristics is subsumed under the concept of need–supply fit (Kristof-Brown et al., 2005). Previous studies have indicated that the fit between motives and values and the opportunity to satisfy them in the workplace predict job satisfaction (Krumm et al., 2013a). Job satisfaction, as a person’s emotional attitude toward his or her job (Locke, 1976) in turn, is highly relevant for job performance (Judge et al., 2001) identification with the organization (de Moura et al., 2009) turnover intention (Van Dick et al., 2004; de Moura et al., 2009), and organizational citizenship behavior (Organ and Ryan, 1995). As an antecedent of several desirable job-related outcomes, job satisfaction as fostered by the congruence between needs and the extent to which the workplace meets these needs constitutes an important concept for researchers and practitioners alike as well as for employers and employees alike. Consequently, job satisfaction as fostered by a need–supply fit holds value in coaching, as one example, because it provides a starting point from which to identify potential sources of low job satisfaction.

### Types of Need–Supply (In)congruence and Job Satisfaction

**FIGURE 1:**
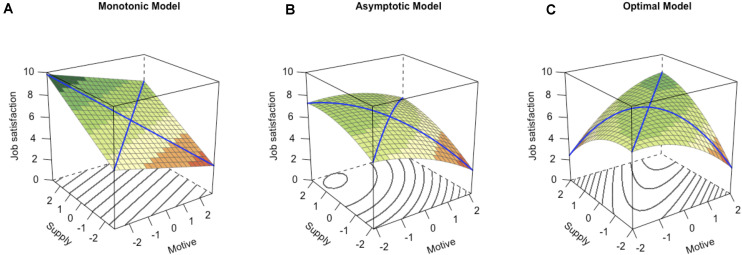
Prototypical response surfaces displaying the **(A)** monotonic, **(B)** asymptotic, and **(C)** optimal models. The vertical axis indicates the level of job satisfaction for different combinations of the motive (right axis) and the supply (left axis). Higher numbers and darker shades of green represent higher levels of job satisfaction. Oversupply (i.e., supply > motive) is displayed along the left edge and undersupply (i.e., supply < motive) is represented along the right edge of each cube.

When relating need–supply fit to job satisfaction, the highest levels of job satisfaction are not necessarily observed when need and supply are exactly congruent. In fact, three different models were introduced to describe this relationship (Harrison, 1978; Edwards, 1996): the monotonic model, the asymptotic model, and the optimal model. Although these models focus on job dissatisfaction, they rely on a unidimensional conceptualization of job (dis)satisfaction ranging from “very dissatisfied” to “very satisfied” (Edwards, 1996). Consequently, reversing their principles allows researchers to apply these models to job satisfaction as the opposite of job dissatisfaction (see Figures 1A–C). Investigations involving actual and desired amounts of certain tasks at work (Edwards, 1996) or larger clusters of values that include work-related motives, values, needs, goals, and interests (Krumm et al., 2013a) have provided support for the three models. We used the monotonic model, the asymptotic model, and the optimal model to derive hypotheses about the effect of oversupply (i.e., an excess in the supply) on job satisfaction. Concerning undersupply (i.e., a shortage in the supply), the effects described in the literature (French et al., 1982) have been less versatile and complex, which is why we did not need different models to derive hypotheses about the impact on job satisfaction. Below, we focus on the effects of oversupply first, before we focus on the consequences of undersupply for job satisfaction.

#### Oversupply

##### The monotonic model

The monotonic model (see Figure 1A) predicts that job satisfaction will be highest for oversupply and that job satisfaction monotonically decreases along the line of incongruence, that is, the blue line in Figure 1A drawn from the left corner of the cube (i.e., supply > motive) to the right corner of the cube (i.e., supply < motive). The processes behind this model are referred to as *conservation* and *carryover* (Edwards, 1996). Conservation applies when an excess in the supply can be saved for a later time, for instance, an accumulation of overtime hours that can be taken off later to satisfy the work value of Leisure Time (i.e., having enough time and energy to spend on one’s private life; Meyer et al., 1998; Krumm et al., 2013b). Carryover comes into effect when an excess in the supply of one motive or need dimension can be used to satisfy a different motive or need dimension; for instance, an excess of Leisure Time (e.g., when working part time) can be used to spend more time with one’s family. A previous study (Krumm et al., 2013a) found a monotonic need–supply fit for value clusters containing, among others, Learning (i.e., to learn novel skills and increase one’s knowledge; Krumm et al., 2013b), Autonomy (i.e., to work independently and be self-responsible; Krumm et al., 2013b) and Appreciation (i.e., to receive esteem; Krumm et al., 2013b). As these motives have shown conceptual overlap with the fundamental motives of Autonomy, Curiosity, and Social Acceptance (see Table 1), we expected the same effect of oversupply on job satisfaction for these motives. Autonomy in particular is arguably associated with a carryover effect, as an excess of autonomy has the potential to introduce desired changes at the workplace (Edwards, 1996). In addition, we expected a carryover effect for dominance and status motives. Because these can be understood as facets of a common power motive (Schönbrodt and Gerstenberg, 2012) we expected that oversupply in one of these motives could be used to satisfy the other motive. For instance, people with high prestige have a certain influential power, and people in a high position have a certain prestige. For Family, Physical Exercise, Food Enjoyment, and Sex, we expect that the vast majority of employed people seek to satisfy these motives not in the workplace but outside their jobs in their leisure time. Thus, we expected that supply for these motives would mainly be found to occur in the form of leisure time so that employees would have enough time to pursue the satisfaction of these motives in their private lives. Because Leisure Time is an important work-related value in itself (Meyer et al., 1998; Krumm et al., 2013b) we expected that an oversupply for Family, Physical Exercise, Food Enjoyment, and Sex could be used to satisfy other motives and values, for instance, Leisure Time itself (i.e., a carryover effect). Finally, for Retention, the conservation effect was already inherent to the definition of the construct (see Table 1). Consequently, we expected that, for an excess in the supply, job satisfaction would increase monotonically.

Hypothesis 1: Job satisfaction will be highest for an oversupply of Social Acceptance, Status, Autonomy, Sex, Retention, Dominance, Family, Physical Exercise, Food Enjoyment, and Curiosity, and these relationships will follow the monotonic model.

##### The asymptotic model

If an excess in the supply of one motive cannot be saved for later use or does not affect the satisfaction of other motives, we predicted an asymptotic relationship between need–supply fit and job satisfaction (see Figure 1B). Here, an excess in the supply would improve job satisfaction only to a small extend beyond the satisfaction of the motive. Consequently, job satisfaction should asymptotically decrease along the line of incongruence. Edwards (1996, p. 295) identified job security as an example of an asymptotic need–supply fit, arguing that the associated supply only applies for a limited period of time, with no harmful or beneficial effects for any excess of supply. Consequently, we expected an asymptotic effect for Safety because it has shown conceptual overlap with the need for job security (Table 1).

Hypothesis 2: Job satisfaction will be highest for an oversupply of Safety, and this relationship will follow the asymptotic model.

##### The optimal model

Finally, the optimal model should apply when oversupply has a negative effect on job satisfaction (see Figure 1C). This is the case when *depletion* or *interference* processes come into effect (Edwards, 1996). Depletion describes the idea that an excess in the supply at one point impedes the future satisfaction of the motive, for instance, an excess in support from a supervisor on one occasion may prevent the employee from receiving additional supervisor support on a later occasion (Edwards, 1996). Interference occurs when an excess in the supply of one motive or need dimension hinders the satisfaction on other motive or need dimensions, for instance, when an excess in job-related travel activity inhibits the desire to spend time with the family. For a value cluster containing, among others, a Helping motive (i.e., providing help to other people; Krumm et al., 2013b) previous results point to an optimal effect. Because Helping has shown conceptual overlap with the fundamental motive of Idealism (see Table 1), we hypothesized the same relationship for this fundamental motive. The underlying process here seems to be interference because, if you need to spend more time and resources providing help to others than would actually be needed to satisfy your motive, this time and these resources cannot be used to pursue the satisfaction of other motives, and job satisfaction would consequently be reduced. We expected the same effect for Structure, Morality, and Revenge. If you need to spend more time and resources structuring your environment, complying with social norms, or retaliating wrongs from others than you actually desire, this time and these resources cannot be used to pursue the satisfaction of other motives. Consequently, we expected job satisfaction to drop for the oversupply of Structure, Morality, and Revenge. Finally, we also expected an interference effect for Social Participation because an oversupply should hinder the satisfaction of the need for privacy and should consequently reduce job satisfaction (Harrison, 1978).

Hypothesis 3: For Idealism, Social Participation, Structure, Morality, and Revenge, job satisfaction will be highest with a congruence between a high supply and a high motive level, and this relationship will follow the optimal model.

#### Undersupply

In addition to the effects of oversupply on job satisfaction described above, Figure 1 indicates that, for undersupply, job satisfaction should be the lowest (French et al., 1982). However, previous research has suggested that this might not hold for all motives. For a value cluster containing, *inter alia*, Autonomy and Learning motives, they found that for a low level of supply, job satisfaction was equally low for all levels of the respective motive (Krumm et al., 2013a). Because the Autonomy and Learning motives have shown strong conceptual overlap with the fundamental motives of Autonomy and Curiosity, respectively, we expected a similar effect of undersupply for these motives.

Hypothesis 4: For all fundamental motives except Autonomy and Curiosity, job satisfaction will be lowest for an undersupply.
Hypothesis 5: For Autonomy and Curiosity, job satisfaction will be lowest at low levels of supply, irrespective of the level of the motive.

### The Present Study

Currently, there is a lack of research that has investigated need–supply fit by considering fine-grained motives in combination with response surface analysis. Traditional approaches to need–supply fit using difference scores have suffered from methodological flaws, including but not limited to a reduction in the reliability of the difference scores compared with the need and supply components they consist of, ambiguity in interpretation of the difference scores, a lack of ways to identify the unique contributions of the need and supply components, and a reduction in the three-dimensional relationship between need, supply, and outcome to the two dimensions, namely, the difference score and the outcome (Edwards, 1994, 2002). Response surface analysis is a powerful tool that can be used to provide a way to overcome these issues by (a) incorporating the need and supply components directly instead of having to compute their difference scores and (b) offering a graphical representation of the three-dimensional relationship between need, supply, and outcome on a detailed level (Edwards, 2002). To extend previous results on the need–supply fit by examining these relations on a more fine-grained level, this study is the first to combine the extensive framework of fundamental motives with the methodological approach of response surface analysis. To this end, we examined five hypotheses, with Hypotheses 1–3 focusing on high levels of job satisfaction and Hypotheses 4 and 5 focusing on low levels of job satisfaction.

By investigating these hypotheses, we were able to identify the combinations of motives and supply that are particularly beneficial to job satisfaction. Vice versa, we gained insight into how over- and undersupply affect job satisfaction. Consequently, we were able to provide detailed insights about which motives are essential for job satisfaction. These motives can be used in coaching to provide guidance to clients about how to change their occupational situation to enhance their job satisfaction and help us understand the specific nature of how the combination of need and supply affects job satisfaction. However, as we tested the monotonic model, the asymptotic model, and the optimal model (Harrison, 1978; Edwards, 1996) we did not expect any congruence effects in a stricter sense, as defined by Humberg et al. (2019, pp. 6–7). In their definition, a congruence effect is present when the response surface is shaped like in the optimal model (Figure 1C), but with a slope of 0 along the line of congruence.

## Materials and Methods

### Participants and Procedure

A total of 723 working people (47% women) from Germany between the ages of 16 and 69 (*M* = 44.13, *SD* = 12.34) participated in the study. Fourteen participants were not native German speakers. Twelve of them reported having very good German language skills, whereas two of them reported good German language skills. A total of 497 (68.7%) participants were employed full-time, whereas 129 (17.8%) participants worked part-time. The remaining participants reported that they were partially retired (*n* = 14, 1.9%), marginally employed (*n* = 50, 6.9%), occasional workers (*n* = 13, 1.8%), or apprentices (*n* = 20, 2.8%). Please consult the online Supplementary Material for a summary of the educational levels of the sample. The sample that was used in this study was a subset comprised of employees from an online sample that was representative of the German population with respect to age and gender. It had previously been used in a different study for a different research question (Dörendahl et al., submitted^1^).

### Measures

#### Fundamental Motives

To assess the fundamental motives, the 16mrs (Dörendahl et al., submitted^1^) was administered. The 16mrs is a questionnaire that was developed and validated using online samples that were representative of the German population. It assesses 16 fundamental motives with three items each. The items were rated on a 6-point Likert scale ranging from 0 (*does not apply at all*) to 5 (*applies completely*). Examples of the items are “I strive to acquire knowledge and make discoveries [Ich strebe nach Wissen und Erkenntnis]” to assess Curiosity, “I like it when others do what I say [Ich mag es, wenn andere tun, was ich sage]” to assess Dominance, and “Sensuality and passion are very important to me [Ich lege großen Wert auf Sinnlichkeit und Leidenschaft]” to assess Sex. The complete list of items is displayed in Dörendahl et al. (submitted)^1^.

#### Supply

We used 16 corresponding items designed to assess supply through the workplace. The items asked the participants about the extent to which their job could satisfy the motives assessed by the 16mrs. For instance, for Social Acceptance, the item asking about supply was “To what extent does your job offer the opportunity to receive acceptance and recognition from other people? [Wie sehr ermöglicht Ihnen Ihre berufliche Tätigkeit, Bestätigung und Anerkennung von anderen zu bekommen?].” The supply items were rated on a 6-point Likert scale ranging from 0 (*not at all*) to 5 (*completely*). The complete list of workplace supply items is available in the online Supplementary Material.

#### Job Satisfaction

Job satisfaction was assessed with a single item that asked participants to rate their job satisfaction. The item was, “How satisfied are you currently with your job? [Wie zufrieden sind Sie derzeit mit Ihrer Arbeit?]” and was rated on a 11-point Likert scale ranging from 0 (*not satisfied at all*) to 10 (*completely satisfied*). Although there has been much discussion about the application of single-item measures, there is a large body of evidence (Gosling et al., 2003) including a meta-analysis by Wanous et al. (1997, p. 250), supporting their reliability for the assessment of job satisfaction.

### Data Analysis

#### Data Preparation

We followed recommendations by Tabachnick and Fidell (2014, pp. 107–108) and identified univariate outliers by examining univariate *z*-score distributions. Cases with |*z*| > 3.29 were further examined using box plots. Eleven cases (1.52%) were identified as outliers because their *z*-scores exceeded the cutoff, and the box plots revealed that they were unattached to the rest of the distribution. These cases were subsequently removed from the data set. Another 36 cases (4.98%) exhibited missing values on the variables used in subsequent analyses. The results of Little’s MCAR test on the item level suggest that the data were not missing completely at random, χ^2^(344) = 415.06, *p* = 0.005. Consequently, we could not deploy missing data imputation and subsequently removed the 36 cases from the data set. Thus, we used 676 cases in the analyses. For this working file, a sensitivity analysis (two-tailed *α* = 0.05, power = 80%) was performed using g^∗^Power (Faul et al., 2007). Results suggested that our sample was sufficiently large enough to detect an *f*^2^ of 0.01, which was even below the cutoff for a small effect (*f*^2^ = 0.02; Cohen, 1988).

#### Response Surface Analysis

To investigate the relations between need–supply fit and job satisfaction, we conducted a series of response surface analyses using the RSA package (Schönbrodt, 2017) for the R environment (R Core Team, 2017). Response surface analysis provides a three-dimensional representation of results obtained from polynomial regression models, which take linear, squared, and interaction effects into account. Consequently, response surface analysis allows for a nuanced examination of relationships between two predictors and an outcome variable (Shanock et al., 2010). To allow for a meaningful interpretation of the results, the motive itself (i.e., the need) and the opportunity to satisfy the respective motive in the workplace (i.e., the supply) were centered on their scale midpoints and entered as predictor variables. Although several methods for centering the predictors exist, we decided to use the scale midpoint because it is not sample dependent (Edwards and Parry, 1993). Job satisfaction constituted the dependent variable. Because job satisfaction was not normally distributed, the resulting models were likely to violate the assumption of normally distributed residuals. To maintain good interpretability of the results, we computed confidence intervals using percentile bootstrapped samples instead of transforming the dependent variable. Percentile bootstrapping was performed with 10,000 bootstrapped samples also using the RSA package.

##### Model selection strategy

Because the full polynomial model is prone to overfitting, we identified the most restrictive models that still fit the data (Schönbrodt, 2016). To this end, we used the RSA package to narrow down candidate models in three steps. First, we investigated the relative model fit in terms of the corrected Akaike Information Criterion (AICc) and retained all models with AICc < 2 because this value indicates practical equivalence (Schönbrodt, 2016). Second, out of the remaining models, we excluded all models with an absolute fit in terms of the comparative fit index (CFI) of <0.95 because such values indicate inadequate fit (Hu and Bentler, 1999). Third, from the pool of the remaining models, we compared the most restrictive model with the next more liberal model using the χ^2^-LR test. If there were several more liberal models with equal numbers of parameters (e.g., for the Dominance motive), we tested the more restrictive model against all of the more liberal models. If the more restrictive model did not fit the data significantly worse, it was retained, but if it was worse, we retained the more liberal model. For Curiosity and Idealism, there was only one candidate model with AICc < 2, so we compared the best model in terms of the AICc against the second-best model, even though its AICc was >2.

## Results

### Descriptives

To provide initial insights into our data, we calculated means, standard deviations, and reliability estimates in terms of alpha and omega. Table 2 displays reliability estimates for the 16mrs as well as means and standard deviations for all measures used in this study. For intercorrelations of the scales, the reader may consult the online Supplementary Material.

**TABLE 2:** Descriptive statistics for the variables used in this study.

Type	Scale	*M*	*SD*	*ω*	*α*
Need	Social acceptance	2.53	0.99	0.63	0.63
	Status	2.12	1.06	0.76	0.76
	Autonomy	3.46	0.82	0.58	0.58
	Sex	2.37	1.09	0.69	0.69
	Retention	3.25	0.95	0.71	0.69
	Dominance	2.37	1.14	0.82	0.81
	Family	3.63	1.06	0.81	0.81
	Physical exercise	2.55	1.32	0.88	0.88
	Food enjoyment	3.23	1.10	0.83	0.82
	Curiosity	3.45	1.01	0.83	0.83
	Safety	3.10	0.98	0.75	0.73
	Idealism	2.93	1.06	0.77	0.76
	Social participation	2.67	1.01	0.77	0.76
	Structure	2.90	1.13	0.80	0.80
	Morality	3.54	0.81	0.64	0.62
	Revenge	2.11	1.22	0.76	0.75
Supply	Social acceptance	3.23	1.32		
	Status	2.52	1.45		
	Autonomy	3.55	1.26		
	Sex	0.38	0.85		
	Retention	2.26	1.56		
	Dominance	2.77	1.48		
	Family	1.77	1.60		
	Physical exercise	1.95	1.68		
	Food enjoyment	0.89	1.41		
	Curiosity	3.68	1.28		
	Safety	2.99	1.39		
	Idealism	2.55	1.77		
	Social participation	3.45	1.36		
	Structure	3.90	1.09		
	Morality	3.40	1.41		
	Revenge	0.78	1.15		
Criterion	Job satisfaction	6.87	2.54		

*M, mean; SD, standard deviation; ω, McDonald’s ω; α, coefficient α.*

### Main Analyses

#### Model Selection Strategy

**FIGURE 2:**
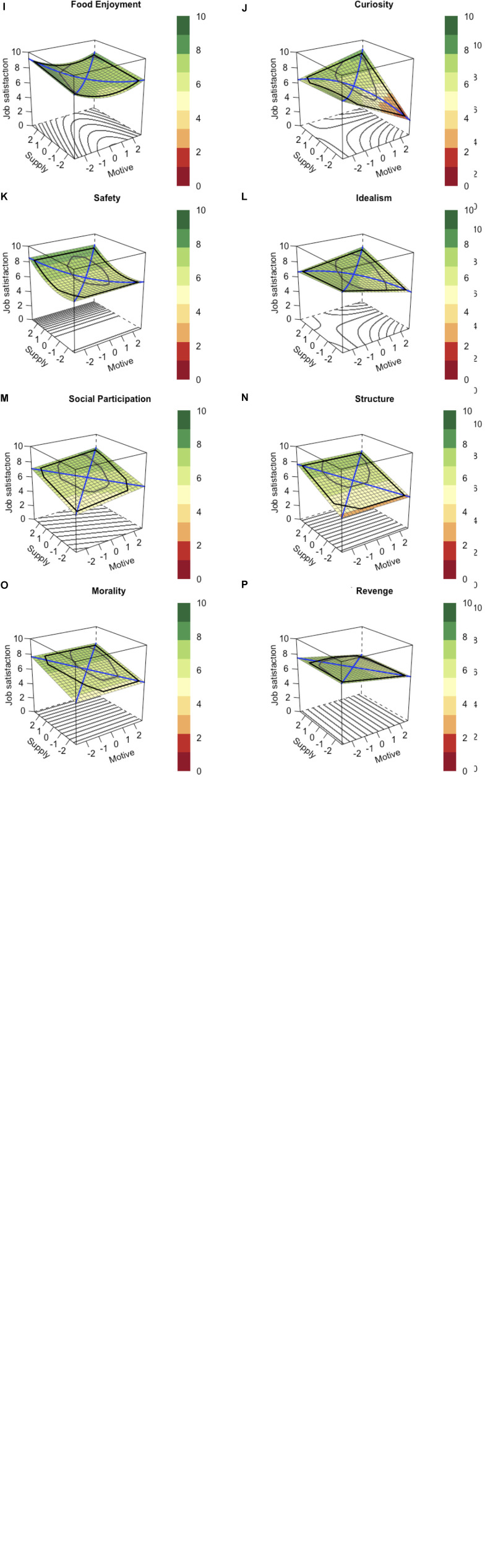
Response surfaces for **(A)** Social Acceptance, **(B)** Status, **(C)** Autonomy, **(D)** Sex, **(E)** Retention, **(F)** Dominance, **(G)** Family, **(H)** Physical Exercise, **(I)** Food Enjoyment, **(J)** Curiosity, **(K)** Safety, **(L)** Idealism, **(M)** Social Participation, **(N)** Structure, **(O)** Morality, and **(P)** Revenge. Higher numbers represent higher levels. The bold line projected on the surface indicates the interpretable region such that only the area inside this line should be interpreted.

The model selection strategy resulted in the 16 models that are displayed in Table 3. The table provides a description of the models with the respective effects included, as well as indices for the relative and absolute fits and explained variance. The selected models explained between 1% (Sex) and 23% (Social Acceptance) of the variance in job satisfaction. For more information about the models, please consult the online Supplemenatray Material or see Schönbrodt (2016, pp. 6–8). The coefficients and parameters for the 16 models are displayed in Table 4. Here, the *b* coefficients are interpreted in the same manner as in linear regression. The *a* parameters deliver additional information about the shape of the response surface. Specifically, *a*_1_ and *a*_2_ describe the slope and the curvature of the line of congruence (diagonal line that goes from the front to the back corner of the response surfaces as displayed in Figures 1, 2). Similarly, *a*_3_ and *a*_4_ represent the slope and the curvature, respectively, of the line of incongruence (diagonal that goes from the left to the right corner of the response surface). A positive slope indicates that the response surface rises along the respective diagonal, whereas a positive curvature signals that the response surface is curved upwards along the respective diagonal. In addition, the so-called ridge of the response surface can be described by the first principal axis, defined by an intercept parameter (*p*_10_) and a slope parameter (*p*_11_). For convex- and saddle-shaped response surfaces, the first principal axis represents the line of greatest upward curvature. For concave response surfaces, the first principal axis represents the line of least downward curvature (Edwards, 2002). However, the first principal axis can only be determined for curved response surfaces.

**TABLE 3:** Results of the model selection process.

Scale	Model	*k*	AICc	ΔAICc	Model weight	CFI	*R*^2^_Adj._	Δχ^2^	*p*_Δχ2_
Social acceptance	**Additive**	4	3,004.77	0.00	0.33	1.00	0.23		
	IA	5	3,005.36	0.59	0.25	1.00	0.23	1.43(1)	0.233
Status	**Additive**	4	3,090.62	0.00	0.40	1.00	0.13		
	SRR	5	3,092.53	1.91	0.16	1.00	0.13	0.11(1)	0.739
	IA	5	3,092.60	1.98	0.15	1.00	0.13	0.04(1)	0.837
Autonomy	**SRSQD**	5	3,064.25	0.00	0.39	1.00	0.16		
	SRRR	6	3,065.89	1.64	0.17	1.00	0.16	0.39(1)	0.535
Sex	**SSQD**	4	3,174.39	0.00	0.34	1.00	0.01		
	SRSQD	5	3,176.20	1.81	0.14	1.00	0.01	0.21(1)	0.647
	SRR	5	3,176.28	1.89	0.13	0.99	0.01	0.13(1)	0.718
Retention	**SRRR**	6	3,157.25	0.00	0.27	0.99	0.04		
	Full	7	3,157.89	0.64	0.20	1.00	0.04	1.39(1)	0.238
Dominance	**Onlyy**	3	3,136.12	1.76	0.10	0.95	0.06		
	Onlyy2	4	3,136.36	1.99	0.09	0.97	0.07	1.77(1)	0.183
	Additive	4	3,134.37	0.00	0.25	1.00	0.07	3.77(1)	0.052
Family	**Onlyy**	3	3,159.58	1.29	0.14	0.95	0.03		
	Onlyy2	4	3,158.29	0.00	0.27	1.00	0.04	3.30(1)	0.069
	Additive	4	3,160.11	1.82	0.11	0.98	0.03	1.48(1)	0.224
Physical exercise	**Onlyy**	3	3,166.92	0.00	0.38	1.00	0.02		
	Onlyy2	4	3,168.61	1.69	0.16	1.00	0.02	0.32(1)	0.571
	Additive	4	3,168.89	1.97	0.14	1.00	0.02	0.04(1)	0.840
Food enjoyment	**SRRR**	6	3,173.52	0.09	0.22	1.00	0.02		
	Full	7	3,175.55	2.12	0.08	1.00	0.01	0.00(1)	0.994
Curiosity	**IA**	5	3,062.56	0.00	0.82	1.00	0.16		
	Full	7	3,066.46	3.90	0.12	1.00	0.16	0.16(2)	0.926
Safety	**Onlyy2**	4	3,132.30	1.62	0.22	0.95	0.07		
	SRRR	6	3,130.68	0.00	0.48	1.00	0.08	5.66(2)	0.059
Idealism	**IA**	5	3,155.17	0.00	0.49	1.00	0.04		
	Full	7	3,159.13	3.96	0.07	1.00	0.04	0.10(2)	0.952
Social participation	Onlyy	3	3,139.22	1.86	0.11	0.98	0.06		
	RR	4	3,139.17	1.82	0.11	1.00	0.06	2.07(1)	0.150
	**Additive**	4	3,137.35	0.00	0.27	1.00	0.06	3.89(1)	0.049
Structure	**Onlyy**	3	3,134.27	0.25	0.19	0.96	0.07		
	Onlyy2	4	3,134.33	0.32	0.18	0.98	0.07	1.95(1)	0.162
	Additive	4	3,135.68	1.67	0.09	0.95	0.07	0.60(1)	0.438
Morality	**Onlyy**	3	3,141.19	1.08	0.20	0.95	0.06		
	Onlyy2	4	3,140.11	0.00	0.35	1.00	0.06	3.10(1)	0.079
Revenge	**Onlyx**	3	3,170.91	0.00	0.40	1.00	0.02		
	Onlyx2	4	3,172.89	1.98	0.15	1.00	0.01	0.04(1)	0.852
	Additive	4	3,172.89	1.98	0.15	1.00	0.01	0.03(1)	0.857

*Boldface, selected model; k, number of parameters estimated in the model; CFI, Comparative fit index; R^2^_Adj._, explained variance adjusted for the number of predictors in the model; IA, interaction model with two linear main effects; full, full polynomial model with two linear main effects, two squared main effects, and an interaction effect; additive, additive main effect model with two linear main effects; onlyy, single linear main effect model with linear main effect of supply; SRRR, shifted and rotated rising ridge model with nonlinear additive and interaction effects; SRSQD, shifted and rotated squared difference model with nonlinear additive and interaction effects; onlyy2, single nonlinear main effect model with squared main effect of supply; onlyx, single linear main effect model with linear main effect of need; SSQD, shifted squared difference model with nonlinear additive and interaction effects. For more information about the models, please see Schönbrodt (2016) pp. 6–8.*

**TABLE 4:** Coefficients and parameters for the 16 RSA models.

	SoA	Sta	Aut	Sex	Ret	Dom	Fam	PhE
*b*_1_/β_1_	**−0.43/−0.17**	**−0.28/−0.12**	**−0.17/−0.06**	**−0.52/−0.22**	**−0.23/−0.08**	–	–	–
	[−0.60, −0.26]	[−0.47, −0.09]	[−0.41, −0.02]	[−0.79, −0.24]	[−0.51, −0.04]			
*b*_2_/β_2_	**0.87/0.46**	**0.67/0.38**	**0.75/0.37**	**0.52/0.17**	**0.33/0.20**	**0.44/0.26**	**0.29/0.18**	**0.23/0.15**
	[0.72, 1.03]	[0.53, 0.81]	[0.54, 0.94]	[0.24, 0.79]	[0.16, 0.49]	[0.30, 0.58]	[0.18, 0.40]	[0.11, 0.34]
*b*_3_/β_3_	–	–	**0.01/0.00**	**0.09/0.06**	**0.00/0.00**	–	–	–
			[0.00, 0.03]	[0.02, 0.17]	[0.00, 0.22]			
*b*_4_/β_4_	–	–	**−0.05/−0.04**	**−0.18/−0.18**	0.03/0.02	–	–	–
			[−0.11, 0.00]	[−0.33, −0.03]	[−0.13, 0.14]			
*b*_5_/β_5_	–	–	**0.10/0.09**	**0.09/0.07**	**0.11/0.10**	–	–	–
			[0.00, 0.21]	[0.02, 0.17]	[0.00, 0.18]			
*p*_10_	–	–	-4.22	–	–	–	–	–
			[−11.21, 2.77]					
*p*_11_	–	–	**−4.34**	–	6.90	–	–	–
			[−8.26, −0.42]		[−19.41, 33.20]			
*a*_1_	**0.44**	**0.39**	**0.57**	–	0.10	–	–	–
	[0.21, 0.67]	[0.20, 0.59]	[0.36, 0.78]		[−0.20, 0.32]			
*a*_2_	–	–	**0.06**	–	**0.14**	–	–	–
			[0.00, 0.16]		[0.01, 0.32]			
*a*_3_	**−1.31**	**−0.95**	**−0.92**	**−1.05**	**−0.55**	–	–	–
	[−1.53, −1.08]	[−1.22, −0.68]	[−1.27, −0.60]	[−1.58, −0.49]	[−0.90, −0.29]			
*a*_4_	–	–	**0.15**	**0.36**	**0.08**	–	–	–
			[0.01, 0.30]	[0.06, 0.66]	[0.00, 0.32]			

	**FoE**	**Cur**	**Saf**	**Ide**	**SoP**	**Str**	**Mor**	**Rev**

*b*_1_/β_1_	−0.12/−0.05	−0.25/−0.10	–	−0.08/−0.03	0.19/0.07	–	–	**−0.27/−0.13**
	[−0.58, 0.17]	[−0.53, 0.04]		[−0.27, 0.11]	[−0.2, 0.39]			[−0.44, −0.1]
*b*_2_/β_2_	**0.21/0.12**	**0.55/0.28**	**0.40/0.22**	**0.22/0.15**	**0.44/0.23**	**0.61/0.26**	**0.44/0.24**	–
	[0.06, 0.52]	[0.33, 0.78]	[0.24, 0.55]	[0.10, 0.34]	[0.29, 0.60]	[0.40, 0.82]	[0.29, 0.58]	
*b*_3_/β_3_	**0.15/0.11**	–	–	–	–	–	–	–
	[0.02, 0.29]							
*b*_4_/β_4_	−0.01/−0.01	**0.24/0.22**	–	**0.13/0.11**	–	–	–	–
	[−0.20, 0.09]	[0.09, 0.40]		[0.03, 0.23]				
*b*_5_/β_5_	**0.00/0.00**	–	**0.14/0.12**	–	–	–	–	–
	[0.00, 0.08]		[0.04, 0.23]					
*p*_10_	–	3.27	–	2.32	–	–	–	–
		[1.09, 5.46]		[−0.28, 4.91]				
*p*_11_	−0.04	–	–	–	–	–	–	–
	[−0.51, 0.43]							
*a*_1_	0.09	0.30	–	0.14	0.63	–	–	–
	[−0.17, 0.34]	[−0.10, 0.71]		[−0.06, 0.34]	[0.40, 0.86]			
*a*_2_	**0.14**	**0.24**	–	**0.13**	–	–	–	–
	[0.01, 0.28]	[0.09, 0.40]		[0.03, 0.23]				
*a*_3_	−0.33	**−0.80**	–	**−0.30**	−0.25	–	–	–
	[−1.06, 0.07]	[−1.10, −0.48]		[−0.55, −0.05]	[−0.54, −0.02]			
*a*_4_	**0.17**	**−0.24**	–	**−0.13**	–	–	–	–
	[0.01, 0.49]	[−0.40, −0.09]		[−0.23, −0.03]				

*Boldface, coefficients and parameters were significant at p < 0.05. 95% confidence intervals based on percentile bootstrapping in brackets; b_1_, motive; b_2_, supply; b_3_, motive^2^; b_4_, motive × supply; b_5_, supply^2^; SoA, Social Acceptance; Sta, Status; Aut, Autonomy; Ret, Retention; Dom, Dominance; Fam, Family; PhE, Physical Exercise; FoE, Food Enjoyment; Cur, Curiosity; Saf, Safety; Ide, Idealism; SoP, Social Participation; Stru, Structure; Mor, Morality; Rev, Revenge.*

#### Oversupply

##### The monotonic model

In Hypothesis 1, we expected that job satisfaction would be highest for an oversupply of Social Acceptance, Status, Autonomy, Sex, Retention, Dominance, Family, Physical Exercise, Food Enjoyment, and Curiosity, all of which were expected to follow a monotonic relationship. The results presented in Table 4 and Figures 2A–J were mixed, with some but not all response surfaces in support of Hypothesis 1. For Social Acceptance (see Figure 2A) and Status (see Figure 2B), the response surfaces strictly complied with the monotonic model (Figure 1A). For these motives, participants reported their highest levels of job satisfaction for oversupply. The response surface sloped downwards in a linear fashion along the line of incongruence. For Autonomy (see Figure 2C), Sex (see Figure 2D), and Retention (see Figure 2E), the response surfaces showed a considerable overlap with the monotonic model (Figure 1A) but also deviated from it in some aspects. For instance, although the highest levels of job satisfaction again occurred for oversupply, there was a squared relation along the line of incongruence. Thus, the higher the level of oversupply, the more negative the slope in the response surface. Consequently, oversupply seemed even more beneficial here compared with Social Acceptance and Status. Although for these three motives, the response surfaces did not strictly comply with the monotonic models, there was still a great deal of overlap with it. By contrast, the response surfaces for Dominance (see Figure 2F), Family (see Figure 2G), Physical Exercise (see Figure 2H), Food Enjoyment (see Figure 2I), and Curiosity (see Figure 2J) clearly deviated from the monotonic model. The results for Dominance, Family, and Physical Exercise suggested that job satisfaction was highest for a high supply, whereas the motive had no impact. For Food Enjoyment, job satisfaction was equally highest for a high supply combined with a low or a high level of the motive, whereas for a high supply and a medium level of the motive, job satisfaction was slightly lower. And finally, for Curiosity, the highest job satisfaction was observed for a combination of high supply and a high motive level, whereas the lowest levels of job satisfaction were observed for oversupply and undersupply. Consequently, the response surface for Curiosity showed a great deal of overlap with the optimal model (Figure 1C) rather than with the monotonic model (Figure 1A). To summarize, for Social Acceptance and Status, the observed need–supply fits strictly complied with the monotonic model and thus supported Hypothesis 1. The response surfaces for Autonomy, Sex, and Retention showed a great deal of overlap with the monotonic model, although they deviated with respect to some features. And finally, the results for Dominance, Family, Physical Exercise, Food Enjoyment, and Curiosity did not support Hypothesis 1 because the respective response surfaces clearly deviated from the monotonic model.

##### The asymptotic model

In Hypothesis 2, for Safety, we expected that job satisfaction would be highest for oversupply and that the measures would have an asymptotic relationship. The results for Safety clearly contradicted Hypothesis 2, as can be seen in Figure 2K, which displays an exponential slope for the supply, whereas the motive had no impact on job satisfaction. Consequently, for Safety, there seemed to be only a relation between job satisfaction and supply, with the squared slope coefficient indicating that for higher levels of supply, the response surface slopes upwards even more.

##### The optimal model

In Hypothesis 3, for Idealism, Social Participation, Structure, Morality, and Revenge, we expected that job satisfaction would be highest for a congruence of a high supply and a high motive level and that the relationships would follow the optimal model. As the results for these motives suggest, none of the response surfaces (see Figures 2L–P) strictly complied with the monotonic model. However, the response surfaces for Idealism (see Figure 2L) and Social Participation (see Figure 2M) showed some overlap with the monotonic model. For both motives, the highest level of job satisfaction was observed for a combination of high supply and a high motive level, whereas job satisfaction was lower for over- and undersupply, respectively. Nevertheless, the response surfaces did not display an optimal relationship. The response surfaces of the remaining motives clearly contradicted Hypothesis 3. For Structure (see Figure 2N) and Morality (see Figure 2O), there was a linear relation for supply only, such that higher levels of job satisfaction were observed at higher supply levels. For Revenge (see Figure 2P), we observed only a linear relation between the motive and job satisfaction, such that lower levels of job satisfaction were observed for higher levels of the motive. In sum, none of the response surfaces strictly complied with Hypothesis 3.

#### Undersupply

In Hypotheses 4 and 5, we focused on predicting the lowest level of job satisfaction. In Hypothesis 4, we expected job satisfaction to be lowest for an undersupply of all motives except for Autonomy and Curiosity. This is the case when the right corner of the response surface representing undersupply shows the lowest values of job satisfaction. The results for Status, Retention, and Idealism supported Hypothesis 4 because the response surfaces (see Figures 2B,E,L) clearly indicated the lowest levels of job satisfaction for an undersupply of each of these motives. In turn, the response surface for Sex (see Figure 2D) does not support Hypothesis 4, as it contained an upward curved line of incongruence. As a consequence, the lowest level of job satisfaction could be observed for a moderate undersupply (i.e., Supply = −1 and Motive = 1), whereas for a strong undersupply (i.e., Supply = −2 and Motive = 2), job satisfaction was slightly higher again. The response surfaces of the other motives did not support Hypothesis 4. For Dominance, Family, Physical Exercise, Safety, Structure, and Morality (see Figures 2F–H,K,N,O), the lowest levels of job satisfaction were observed for a low supply irrespective of the motive level. The opposite result was observed for Revenge (see Figure 2P). Here, job satisfaction was lowest for high levels of the motive, irrespective of the level of supply. For Social Participation (see Figure 2M), the lowest level of supply was observed for a combination of low supply and a low level of the motive. And finally, for Food Enjoyment (see Figure 2I), the lowest levels of job satisfaction occurred for a combination of low supply and a medium level of the motive. In sum, the response surfaces for Status, Retention, and Idealism support Hypothesis 4, as they clearly showed the hypothesized shape. The response surfaces for the remaining motives contradicted Hypothesis 4.

In Hypothesis 5, we expected that for Autonomy and Curiosity, job satisfaction would be lowest for low levels of supply, irrespective of the level of the motive. The response surface for Autonomy (see Figure 2C) supported Hypothesis 5 because job satisfaction was lowest for a low supply, irrespective of the level of the motive. The response surface for Curiosity (see Figure 2J) did not support Hypothesis 5 because the lowest level of job satisfaction was observed for undersupply, whereas job satisfaction for a combination of a low supply and a low motive level was higher. In sum, the results for Autonomy supported Hypothesis 5, whereas the results for Curiosity did not.

## Discussion

Need–supply fit is a psychological concept of high relevance, particularly for employees and consequently also for employers. To provide detailed insights into how the satisfaction of explicit motives in the workplace contributes to job satisfaction, we conducted a series of response surface analyses, using the comprehensive yet fine-grained framework of fundamental motives. To this end, we investigated five hypotheses, based on the need–supply fit literature (Edwards, 1996) and previous findings (French et al., 1982; Edwards, 1996; Krumm et al., 2013a). With respect to predicting high levels of job satisfaction, some of the response surfaces supported Hypotheses 1 and 3 involving oversupply, while others deviated from the hypothesized shape. Hypothesis 2, involving an oversupply of Safety, was not supported. Concerning low levels of job satisfaction, also some of the response surfaces supported Hypotheses 4 and 5, while some results contradicted them.

Beyond the hypotheses, the results have important implications for avoiding dissatisfaction and enhancing satisfaction. With respect to oversupply, the results for Social Acceptance, Status, Autonomy, Sex, and Retention suggest that an excess in supply can be saved for use at a later time (i.e., conservation) or to satisfy a different motive or need dimension (i.e., carryover). To clarify which of these processes comes into effect for which motive, future investigations should focus on identifying the exact nature of the supply and other motives or needs for which the supply can be used. A slightly negative result of oversupply was observed for Curiosity, Idealism, and Social Participation. Although the response surfaces for these models only remotely followed the optimal model (Figure 1C), the levels of job satisfaction for oversupply were still lower compared with congruence between high motive and high supply levels. Consequently, an excess in the supply of these motives may impede the future satisfaction of the same motive (i.e., depletion) or hinder the satisfaction of other motive or need dimensions (i.e., interference). Again, future investigations should focus on identifying the exact nature of the supply to clarify which of the two processes comes into effect for the respective motives. Beyond the results associated with the monotonic and optimal models, we found another group of motives (i.e., Dominance, Family, Physical Exercise, Safety, Structure, and Morality) that showed similar shapes in their response surfaces but no overlap with any of the three theoretical models. For these dimensions, higher job satisfaction was observed for a higher supply, irrespective of the strength of the respective motive. A potential explanation could be that conservation/carryover effects and depletion/interference effects cancel each other out, resulting in neither an overly positive nor a negative effect of oversupply on job satisfaction. For instance, an oversupply in dominance through a promotion beyond one’s aspirations might also satisfy the status motive (i.e., carryover). By contrast, a higher position in a company is likely to be associated with a higher workload that may consequently impede the satisfaction of the need for Leisure Time (i.e., interference). And finally, Food Enjoyment and Revenge each showed unique response surfaces that also differed considerably from the theoretical models.

Concerning the prediction of low levels of job satisfaction, two types of results stood out. For a group of fundamental motives including Social Acceptance, Status, Sex, Retention, Curiosity, and Idealism, the lowest levels of job satisfaction were observed for undersupply. This finding is in line with previous research (French et al., 1982). Deviating from this, for the fundamental motives Autonomy, Dominance, Family, Physical Exercise, Safety, Structure, and Morality, the lowest levels of job satisfaction occurred for low levels of supply, irrespective of the level of the motive. Concerning the amount of explained variance (i.e., between 1% for Sex and 23% for Social Acceptance), the need–supply fit for the fundamental motives explained less variance compared with larger clusters of values that are specifically tailored to the work place (Krumm et al., 2013a).

As the monotonic model, the asymptotic model, and the optimal model (Harrison, 1978; Edwards, 1996) significantly deviate from the strict definition of congruence by Humberg et al. (2019, pp. 6–7), we explicitly did not expect any strict congruence effects to occur. However, it is worth mentioning for reasons of clarity that, indeed, none of the models represented a strict congruence effect. In the presence of a strict congruence effect, all participants whose motive levels are exactly met by the supply levels of their job would equally report the highest levels of job satisfaction. However, as our results show, this was not the case for any of the response surfaces. Either there was significant slope along the line of congruence (e.g., Social Acceptance), a significant curvature along the line of congruence (e.g., Idealism), or both (e.g., Autonomy). As a result, the reported levels of job satisfaction differ between points on the line of congruence.

### Implications

Our findings have several theoretical and practical implications. The results support the validity of need–supply fit with fundamental motives as a predictor for job satisfaction. In terms of the amount of explained variance, Curiosity, Social Acceptance, Autonomy, and Status seem to be most important out of the 16 fundamental motives. Thus, the results further support the assumption that the 16 fundamental motives are important in a variety of domains in people’s everyday lives (Havercamp, 1998; Reiss and Havercamp, 1998). Previous research has focused on the validity of the 16 fundamental motives in life domains such as interpersonal relations (Engel et al., 2002; Man et al., 2006) and school achievement (Froiland et al., 2015). The present study further adds to these results by providing support for the validity of the 16 fundamental motives in the work domain.

With respect to practical implications, the results underscore the importance of using fine-grained motives when it comes to coaching and career development based on the fit between employee characteristics and the characteristics of the job itself. Concerning this matter, we were able to demonstrate that fine-grained motives that belong to the same cluster of work values show need–supply fit relationships with job satisfaction that differ considerably from one another. For instance, results involving the work values cluster of intrinsic growth (Krumm et al., 2013a) imply that the relationship of Curiosity and Autonomy, which belong to the same cluster, and job satisfaction can be described by the monotonic model (Figure 1). However, the results presented in this study now suggest that only Autonomy shows an approximately monotonic effect, whereas the need–supply fit for Curiosity can be better described by the optimal model (Figure 1C). Consequently, the implications are quite different. Whereas for Autonomy, oversupply is the desirable state with respect to enhancing job satisfaction to a maximal level, oversupply would be suboptimal for Curiosity. Here, a congruence between a high supply level and a high motive level is most beneficial for job satisfaction.

In sum, the satisfaction of fundamental motives has a considerable impact on job satisfaction. Especially models including Curiosity, Social Acceptance, Autonomy, and Status are able to explain a substantial amount of variance in job satisfaction. Thus, the need–supply fit for these motives can be assessed in coaching and career development to identify potential sources of a client’s dissatisfaction at work. Consequently, the identification of such misfits in coaching and career development provides a starting point for clients with respect to how to change their occupational situation to ultimately enhance their job satisfaction. Here, fundamental motives come with the advantage of a low level of abstraction, which allows a straightforward interpretation for clients. In addition, the models that had significant effects only for supply provided useful practical implications. For these dimensions, the prediction of job satisfaction was independent of the person’s standing on the corresponding motive. Consequently, an increase in the supply may benefit all employees equally by increasing their job satisfaction. Specifically, the supply of Dominance, Morality, Structure, and Safety explained substantial variance and consequently constituted starting points for employers to increase their employees’ job satisfaction.

### Strengths and Limitations

The present study has several methodological strengths, including a large sample that was a subset of an online sample that was representative of the German population with respect to age and gender and the application of response surface analyses, which provide a detailed examination of how combinations of need and supply are related to job satisfaction. However, there are also limitations that should be addressed in future research.

First, due to time restrictions, we used economical single-item indicators to assess job satisfaction and the need domains. Although previous research has suggested that the reliability of single-item indicators is sufficiently high (e.g., Wanous et al., 1997; Robins et al., 2001; Nagy, 2002; Gosling et al., 2003; Hoeppner et al., 2011; Lucas and Donnellan, 2012) the constructs might not be covered in a broad enough way from a conceptual view. To validate the results obtained in this study, future studies should use multi-indicator scales to assess the supply domain and job satisfaction. For job satisfaction, established measures can be used, for instance, the overall job satisfaction scale (Judge et al., 1998). For the supply domains, corresponding to the fundamental motives, a multi-item inventory would need to be developed either from scratch or based on the items used in this study.

Second, for the score from the Autonomy scale, reliability estimates of 0.58 (both *α* and *ω*) were slightly below the recommended cutoff of 0.60 (Sijtsma and Molenaar, 2002). Consequently, the effect sizes and amount of explained variance might be attenuated for the respective model, and the results should be interpreted with caution. Future research might rely on a more reliable scale to assess the Autonomy domain. Although the remaining measures exceeded the reliability lower bound of 0.60 (see Table 2), some effects in the models might not have turned out to be significant due to measurement error (Su et al., 2019). Therefore, future research might utilize the latent moderated structural equations (LMS) approach to correct for lack of reliability in the need, supply, and outcome measures (Su et al., 2019).

Third, the large sample used in this study was cross-sectional. Consequently, the results obtained from these data do not allow for any kind of causal inferences to be made with respect to the relationship between need–supply fit of fundamental motives and job satisfaction. Future studies may therefore wish to apply longitudinal designs to gather further support for the direction of the relationship, that is, need–supply fit influences job satisfaction and not vice versa.

## Conclusion

The results underscore the importance of fundamental motives for job satisfaction. Consequently, fundamental motives can be used in coaching and career development to help uncover sources of low satisfaction and provide guidance to clients in how to change their occupational situation to enhance their job satisfaction. As an advantage, fundamental motives provide a very low level of abstraction, which is straightforward for clients to understand.

## Data Availability Statement

The datasets generated for this study are available on request to the corresponding author.

## Ethics Statement

Participants for this study were sampled by the private survey institute forsa main (forsa.de), based in Germany. This independent institute conducts surveys for research, political, and state institutions as well as for companies. Forsa is a member of ESOMAR (esomar.org), which ensures that data collection, storage, and processing are conducted in a safe and ethical manner. The data were fully anonymized by forsa main before all authors had access to them. The participants were informed that the results of this survey would potentially be used for academic publications.

## Author Contributions

JD contributed to the conceptualization, formal analysis, and writing (original draft preparation). CN contributed to the supervision and writing (review and editing). SG contributed to the project administration and writing (review and editing). All authors contributed to the article and approved the submitted version. The authors would also like to acknowledge the contributions of Christoph J. Kemper, Thomas Staller, Peter Boltersdorf, and John Delnoy, who offered helpful ideas and suggestions on an earlier version of this article.

## Conflict of Interest

The authors declare that the research was conducted in the absence of any commercial or financial relationships that could be construed as a potential conflict of interest.

## Footnotes

1 Dörendahl, J., Greiff, S., and Niepel, C. (submitted). Assessing 16 fundamental motives with fewer than 50 items: development and validation of the 16 motives research scales (16mrs).
