# Supplementary material for: Actually Getting Some Satisfaction on the Job: Need–Supply Fit of Fundamental Motives at Work
Source: Front Psychol. 2020 Aug 12;11:1740. doi: 10.3389/fpsyg.2020.01740 (PMC7437360; doi:10.3389/fpsyg.2020.01740)
Supplement: Supplementary file 1 [file Data_Sheet_1.docx]

*Online Supplementary Material for:*

**Actually Getting Some Satisfaction on the Job: Need-Supply Fit of Fundamental Motives at Work**

Note: This supplementary material is intended to appear only on the website linked to the article, and is not intended for the printed article.

**Table of Content**

[Table 1: Items Used to Assess the Supply through the Workplace for Each Fundamental Motive 2](#_Toc1058860)

[Table 2: Educational levels 4](#_Toc1058919)

[Table 3: Spearman Rank Correlations for the 16mrs Scales, the Items Assessing Supply through the Workplace, and Job Satisfaction 5](#_Toc1058930)

[Table 4: Spearman Rank Correlations for the 16mrs Scales 7](#_Toc1058935)

[Table 5: Spearman Rank Correlations for the Items Assessing Supply through the Workplace 8](#_Toc1058950)

[Table 6: Effects Included in the Selected Model for Each Motive 9](#_Toc1058951)

[References 11](#_Toc1058935)

Table 1

*Items Used to Assess the Supply through the Workplace for Each Fundamental Motive*

| Scale | Item |
| --- | --- |
| Curiosity | To what extent does your job offer the opportunity to challenge your mind and gain knowledge? |
| Social Acceptance | To what extent does your job offer the opportunity to receive acceptance and recognition from other people? |
| Dominance | To what extent does your job offer the opportunity to influence other people? |
| Status | To what extent does your job offer the opportunity to increase your social prestige? |
| Retention | To what extent does your job offer the opportunity to economize stocks? |
| Autonomy | To what extent does your job offer the opportunity to work autonomously or be independent? |
| Social Participation | To what extent does your job offer the opportunity to establish cordial relationships with other people and maintain them? |
| Morality | To what extent does your job offer the opportunity to act in a moral way and obey the rules and laws of society? |
| Idealism | To what extent does your job offer the opportunity to support people in need? |
| Structure | To what extent does your job offer the opportunity to structure things or processes? |
| Safety | To what extent does your job offer the opportunity to live a quiet and safe life? |
| Revenge | To what extent does your job offer the opportunity to get back at people who offended you? |
| Physical Exercise | To what extent does your job offer the opportunity to be physically active? |

*Table 1 (continued)*

| Food Enjoyment | To what extent does your job offer the opportunity to have enjoyable experiences with food? |
| --- | --- |
| Family | To what extent does your job contribute to your ability to spend time with your family? |
| Sex | To what extent does your job contribute to your ability to have an active and fulfilling sexual life? |

Table 2

Educational levels

|  | | n | | % |
| --- | --- | --- | --- | --- |
| No school leaving qualification | 3 | | 0.4 | |
| Degree from lower track school | 170 | | 23.5 | |
| Degree from intermediate track school | 262 | | 36.2 | |
| Degree from higher track school | 288 | | 39.8 | |

*Note.* The educational levels reported in this table refer to the German

school system, which typically distributes students to one of three different tracks (lower, intermediate, and higher tracks) for secondary education on the basis of their scholastic achievement inelementary school. In this system, a degree from a higher track school is required for university entrance.

Table 3

*Spearman Rank Correlations for the 16mrs Scales, the Items Assessing Supply through the Workplace, and Job Satisfaction*

|  |  | Supply | | | | | | | | | | | | | | | |  |
| --- | --- | --- | --- | --- | --- | --- | --- | --- | --- | --- | --- | --- | --- | --- | --- | --- | --- | --- |
|  | Scale | Cur | SoA | Dom | Sta | Ret | Aut | SoP | Mor | Ide | Stru | Saf | Rev | PhE | FoE | Fam | Sex | JoS |
| Motive | Cur | .35 | .25 | .24 | .23 | .17 | .22 | .11 | .16 | .09 | .21 | .04 | .07 | .01 | -.02 | -.05 | -.01 | .14 |
|  | SoA | -.00 | .02 | .08 | .07 | .06 | -.06 | .05 | .01 | .02 | .05 | .04 | .17 | .02 | -.01 | -.01 | .03 | -.16 |
|  | Dom | .16 | .17 | .35 | .26 | .14 | .10 | .14 | .09 | .04 | .16 | .07 | .27 | .07 | .10 | -.00 | .08 | .00 |
|  | Sta | .15 | .18 | .28 | .34 | .16 | .07 | .14 | .08 | .08 | .15 | .07 | .28 | .07 | .09 | .01 | .10 | -.01 |
|  | Ret | .06 | .00 | .08 | .03 | .26 | .04 | .00 | .05 | -.02 | .11 | .06 | .01 | .07 | -.01 | -.02 | .02 | -.05 |
|  | Aut | .16 | .11 | .08 | .06 | .06 | .14 | .11 | .16 | .04 | .18 | .06 | .04 | .06 | -.10 | -.11 | -.06 | .00 |
|  | SoP | .11 | .18 | .11 | .22 | .05 | .03 | .20 | .07 | .10 | .07 | .06 | -.02 | .10 | .14 | .14 | .10 | .11 |
|  | Mor | .15 | .13 | .05 | .12 | .13 | .16 | .10 | .19 | .09 | .20 | .12 | -.04 | .05 | -.10 | .05 | -.08 | .08 |
|  | Ide | .15 | .13 | .15 | .14 | .04 | .08 | .16 | .18 | .25 | .09 | -.01 | -.06 | .09 | .04 | .09 | -.04 | .02 |
|  | Stru | .03 | .02 | .05 | .06 | .11 | .06 | .05 | .04 | -.00 | .19 | .12 | .02 | .07 | -.04 | -.02 | -.07 | .01 |
|  | Saf | -.05 | .00 | -.06 | -.04 | .06 | .04 | .03 | .05 | .01 | .07 | .06 | -.03 | .07 | -.05 | -.06-. | -.04 | -.01 |
|  | Rev | .06 | .03 | .12 | .08 | .12 | .06 | .02 | .00 | -.06 | .05 | .01 | .31 | .03 | .01 | -.03 | .04 | -.12 |
|  | PhE | .02 | .01 | .04 | .02 | .06 | .03 | .03 | .01 | .02 | .01 | .01 | .01 | .16 | .05 | .06 | .13 | .03 |
|  | FoE | .10 | .06 | .03 | .05 | .11 | .05 | .11 | .11 | -.02 | .08 | -.01 | -.06 | .09 | .16 | .10 | -.01 | .06 |
|  | Fam | .11 | .12 | .09 | .09 | .04 | .11 | .13 | .15 | .07 | .14 | .06 | -.03 | .07 | -.02 | .19 | -.05 | .10 |
|  | Sex | .12 | .08 | .16 | .12 | .06 | .05 | .15 | .06 | -.00 | .08 | .02 | .11 | .10 | .18 | -.04 | .20 | -.03 |
|  | JoS | .36 | .46 | .26 | .31 | .15 | .41 | .26 | .24 | .17 | .26 | .26 | -.04 | .15 | .09 | .16 | .01 | – |

*Note.* 16mrs scales are displayed in the rows; Items for assessing supply are displayed in columns; Cur = Curiosity; SoA = Social Acceptance; Dom = Dominance; Sta = Status; Ret = Retention; Aut = Autonomy; SoP = Social Participation; Mor = Morality; Ide = Idealism; Stru = Structure; Saf = Safety; Rev = Revenge; PhE = Physical Exercise; FoE = Food Enjoyment; Fam = Family; JoS = Job Satisfaction; |*r|* > .07, *p* < .05; |*r|* > .10, *p* < .01; |*r|* > .12, *p* < .001.

| Table 4  *Spearman Rank Correlations for the 16mrs Scales* | | | | | | | | | | | | | | | | | | | | | | | | | | | | | | | | | |
| --- | --- | --- | --- | --- | --- | --- | --- | --- | --- | --- | --- | --- | --- | --- | --- | --- | --- | --- | --- | --- | --- | --- | --- | --- | --- | --- | --- | --- | --- | --- | --- | --- | --- |
|  | | 1 | | 2 | | 3 | | 4 | | 5 | | 6 | | 7 | | 8 | | 9 | | 10 | | 11 | | 12 | | 13 | | 14 | | 15 | | 16 | |
| Curiosity (1) |  | — |  |  |  |  |  |  |  |  |  |  |  |  |  |  |  |  |  |  |  |  |  |  |  |  |  |  |  |  |  |  |  |
| Social Acceptance (2) |  | .08 |  | — |  |  |  |  |  |  |  |  |  |  |  |  |  |  |  |  |  |  |  |  |  |  |  |  |  |  |  |  |  |
| Dominance (3) |  | .31 |  | .30 |  | — |  |  |  |  |  |  |  |  |  |  |  |  |  |  |  |  |  |  |  |  |  |  |  |  |  |  |  |
| Status (4) |  | .33 |  | .40 |  | .72 |  | — |  |  |  |  |  |  |  |  |  |  |  |  |  |  |  |  |  |  |  |  |  |  |  |  |  |
| Retention (5) |  | .18 |  | .21 |  | .11 |  | .17 |  | — |  |  |  |  |  |  |  |  |  |  |  |  |  |  |  |  |  |  |  |  |  |  |  |
| Autonomy (6) |  | .36 |  | .19 |  | .21 |  | .20 |  | .25 |  | — |  |  |  |  |  |  |  |  |  |  |  |  |  |  |  |  |  |  |  |  |  |
| Social Participation (7) |  | .19 |  | .06 |  | .28 |  | .33 |  | .10 |  | .07 |  | — |  |  |  |  |  |  |  |  |  |  |  |  |  |  |  |  |  |  |  |
| Morality (8) |  | .29 |  | .29 |  | .12 |  | .19 |  | .33 |  | .30 |  | .20 |  | — |  |  |  |  |  |  |  |  |  |  |  |  |  |  |  |  |  |
| Idealism (9) |  | .35 |  | .08 |  | .14 |  | .17 |  | .07 |  | .16 |  | .36 |  | .38 |  | — |  |  |  |  |  |  |  |  |  |  |  |  |  |  |  |
| Structure (10) |  | .09 |  | .27 |  | .19 |  | .20 |  | .35 |  | .24 |  | .14 |  | .35 |  | .05 |  | — |  |  |  |  |  |  |  |  |  |  |  |  |  |
| Safety (11) |  | .01 |  | .50 |  | .06 |  | .16 |  | .30 |  | .25 |  | .02 |  | .44 |  | .05 |  | .33 |  | — |  |  |  |  |  |  |  |  |  |  |  |
| Revenge (12) |  | .10 |  | .25 |  | .34 |  | .31 |  | .10 |  | .28 |  | -.02 |  | .01 |  | -.01 |  | .05 |  | .14 |  | — |  |  |  |  |  |  |  |  |  |
| Physical Exercise (13) |  | .11 |  | .02 |  | .12 |  | .14 |  | .23 |  | .10 |  | .30 |  | .17 |  | .20 |  | .21 |  | .01 |  | -.03 |  | — |  |  |  |  |  |  |  |
| Food Enjoyment (14) |  | .23 |  | -.04 |  | .13 |  | .13 |  | .17 |  | .19 |  | .32 |  | .21 |  | .15 |  | .14 |  | .06 |  | .01 |  | .21 |  | — |  |  |  |  |  |
| Family (15) |  | .06 |  | .19 |  | .16 |  | .17 |  | .15 |  | .17 |  | .29 |  | .35 |  | .21 |  | .23 |  | .30 |  | .10 |  | .10 |  | .23 |  | — |  |  |  |
| Sex (16) |  | .17 |  | .19 |  | .31 |  | .37 |  | .04 |  | .12 |  | .34 |  | .08 |  | .18 |  | .12 |  | .04 |  | .21 |  | .20 |  | .18 |  | .11 |  | — |  |

*Note.* |*r|* > .07, *p* < .05; |*r|* > .10, *p* < .01; |*r|* > .12, *p* < .001.

| Table 5  *Spearman Rank Correlations for the Items Assessing Supply through the Workplace* | | | | | | | | | | | | | | | | | | | | | | | | | | | | | | | | | |
| --- | --- | --- | --- | --- | --- | --- | --- | --- | --- | --- | --- | --- | --- | --- | --- | --- | --- | --- | --- | --- | --- | --- | --- | --- | --- | --- | --- | --- | --- | --- | --- | --- | --- |
|  | | 1 | | 2 | | 3 | | 4 | | 5 | | 6 | | 7 | | 8 | | 9 | | 10 | | 11 | | 12 | | 13 | | 14 | | 15 | | 16 | |
| Curiosity (1) |  | — |  |  |  |  |  |  |  |  |  |  |  |  |  |  |  |  |  |  |  |  |  |  |  |  |  |  |  |  |  |  |  |
| Social Acceptance (2) |  | .47 |  | — |  |  |  |  |  |  |  |  |  |  |  |  |  |  |  |  |  |  |  |  |  |  |  |  |  |  |  |  |  |
| Dominance (3) |  | .42 |  | .43 |  | — |  |  |  |  |  |  |  |  |  |  |  |  |  |  |  |  |  |  |  |  |  |  |  |  |  |  |  |
| Status (4) |  | .41 |  | .56 |  | .50 |  | — |  |  |  |  |  |  |  |  |  |  |  |  |  |  |  |  |  |  |  |  |  |  |  |  |  |
| Retention (5) |  | .22 |  | .19 |  | .25 |  | .26 |  | — |  |  |  |  |  |  |  |  |  |  |  |  |  |  |  |  |  |  |  |  |  |  |  |
| Autonomy (6) |  | .47 |  | .43 |  | .33 |  | .35 |  | .21 |  | — |  |  |  |  |  |  |  |  |  |  |  |  |  |  |  |  |  |  |  |  |  |
| Social participation (7) |  | .31 |  | .48 |  | .43 |  | .38 |  | .07 |  | .20 |  | — |  |  |  |  |  |  |  |  |  |  |  |  |  |  |  |  |  |  |  |
| Morality (8) |  | .27 |  | .36 |  | .37 |  | .34 |  | .20 |  | .30 |  | .37 |  | — |  |  |  |  |  |  |  |  |  |  |  |  |  |  |  |  |  |
| Idealism (9) |  | .23 |  | .33 |  | .38 |  | .38 |  | .06 |  | .16 |  | .33 |  | .36 |  | — |  |  |  |  |  |  |  |  |  |  |  |  |  |  |  |
| Structure (10) |  | .54 |  | .42 |  | .35 |  | .37 |  | .20 |  | .44 |  | .27 |  | .36 |  | .19 |  | — |  |  |  |  |  |  |  |  |  |  |  |  |  |
| Safety (11) |  | .14 |  | .13 |  | .07 |  | .14 |  | .25 |  | .18 |  | .09 |  | .16 |  | -.03 |  | .16 |  | — |  |  |  |  |  |  |  |  |  |  |  |
| Revenge (12) |  | -.00 |  | .11 |  | .28 |  | .19 |  | .19 |  | .02 |  | .09 |  | -.04 |  | .07 |  | .04 |  | .00 |  | — |  |  |  |  |  |  |  |  |  |
| Physical Exercise (13) |  | .00 |  | .11 |  | .19 |  | .14 |  | .15 |  | .06 |  | .19 |  | .05 |  | .17 |  | .01 |  | .02 |  | .08 |  | — |  |  |  |  |  |  |  |
| Food Enjoyment (14) |  | .03 |  | .13 |  | .14 |  | .18 |  | .28 |  | .03 |  | .09 |  | -.00 |  | .07 |  | .01 |  | .01 |  | .19 |  | .26 |  | — |  |  |  |  |  |
| Family (15) |  | .09 |  | .05 |  | .06 |  | .12 |  | .14 |  | .11 |  | .11 |  | .14 |  | .10 |  | .05 |  | .24 |  | .03 |  | .11 |  | .23 |  | — |  |  |  |
| Sex (16) |  | -.04 |  | .02 |  | .04 |  | .08 |  | .11 |  | -.05 |  | -.02 |  | -.02 |  | .05 |  | -.10 |  | .02 |  | .23 |  | .20 |  | .37 |  | .25 |  | — |  |

*Note.* |*r|* > .07, *p* < .05; |*r|* > .10, *p* < .01; |*r|* > .12, *p* < .001.

Table 6

*Effects Included in the Selected Model for Each Motive*

| Motive | Model | Main effect motive | Main effect supply | Squared effect motive | Squared effect supply | Interaction effect |
| --- | --- | --- | --- | --- | --- | --- |
| Cur | IA | Included | Included | – | – | Included |
| SoA | additive | Included | Included | – | – | – |
| Dom | onlyy | – | Included | – | – | – |
| Sta | additive | Included | Included | – | – | – |
| Ret | SRRR | Included | Included | Included | Included | Included |
| Aut | SRSQD | Included | Included | Included | Included | Included |
| SoP | additive | Included | Included | – | – | – |
| Mor | onlyy | – | Included | – | – | – |
| Ide | IA | Included | Included | – | – | Included |
| Stru | onlyy | – | Included | – | – | – |
| Saf | onlyy2 | – | Included | – | Included | – |
| Rev | onlyx | Included | – | – | – | – |
| PhE | onlyy | – | Included | – | – | – |
| FoE | SRRR | Included | Included | Included | Included | Included |
| Fam | onlyy | – | Included | – | – | – |
| Sex | SSQD | Included | Included | Included | Included | Included |

*Note.* Cur = Curiosity; SoA = Social Acceptance; Dom = Dominance; Sta = Status; Ret = Retention; Aut = Autonomy; SoP = Social Participation; Mor = Morality; Ide = Idealism; Stru = Structure; Saf = Safety; Rev = Revenge; PhE = Physical Exercise; FoE = Food Enjoyment; Fam = Family; IA = Interaction model with two linear main effects; full = full polynomial model with two linear main effects, two squared main effects, and an interaction effect; additive = Additive main effect model with two linear main effects; onlyy = Single linear main effect model with linear main effect of supply; SRRR = Shifted and rotated rising ridge model with nonlinear additive and interaction effects; SRSQD = Shifted and rotated squared difference model with nonlinear additive and interaction effects; onlyy2 = Single nonlinear main effect model with squared main effect of supply; onlyx = Single linear main effect model with linear main effect of need; SSQD = Shifted squared difference model with nonlinear additive and interaction effects. For more information about the models, please see Schönbrodt (2016, pp. 6-8) .

References

Schönbrodt, F. D. (2016). Testing fit patterns with polynomial regression models. *Manuscript Submitted for Publication*. Retrieved from osf.io/3889z
